# Monensin inhibits cell proliferation and tumor growth of chemo-resistant pancreatic cancer cells by targeting the EGFR signaling pathway

**DOI:** 10.1038/s41598-018-36214-5

**Published:** 2018-12-17

**Authors:** Xin Wang, Xingye Wu, Zhonglin Zhang, Chao Ma, Tingting Wu, Shengli Tang, Zongyue Zeng, Shifeng Huang, Cheng Gong, Chengfu Yuan, Linghuan Zhang, Yixiao Feng, Bo Huang, Wei Liu, Bo Zhang, Yi Shen, Wenping Luo, Xi Wang, Bo Liu, Yan Lei, Zhenyu Ye, Ling Zhao, Daigui Cao, Lijuan Yang, Xian Chen, Rex C. Haydon, Hue H. Luu, Bing Peng, Xubao Liu, Tong-Chuan He

**Affiliations:** 1 Department of Pancreatic Surgery, West China Hospital of Sichuan University, Chengdu, 610041 China; 2 Molecular Oncology Laboratory, Department of Orthopaedic Surgery and Rehabilitation Medicine, The University of Chicago Medical Center, Chicago, IL 60637 USA; 3 Departments of Surgery, Clinical Laboratory Medicine, Orthopaedic Surgery, Plastic Surgery and Burn, Otolaryngology, Head and Neck Surgery, and Obstetrics and Gynecology, the First Affiliated Hospital of Chongqing Medical University, Chongqing, 400016 China; 4 Departments of Hepatobiliary & Pancreatic Surgery, Neurosurgery, and Otolaryngology, Head and Neck Surgery, the Affiliated Zhongnan Hospital of Wuhan University, Wuhan, 430071 China; 5 Ministry of Education Key Laboratory of Diagnostic Medicine and School of Laboratory Medicine, and the Affiliated Hospitals of Chongqing Medical University, Chongqing, 400016 China; 6 Department of Biochemistry and Molecular Biology, China Three Gorges University School of Medicine, Yichang, 443002 China; 7 Department of Clinical Laboratory Medicine, the Second Affiliated Hospital of Nanchang University, Nanchang, 330031 China; 8 Key Laboratory of Orthopaedic Surgery of Gansu Province, and the Departments of Orthopaedic Surgery and Obstetrics and Gynecology, the First and Second Hospitals of Lanzhou University, Lanzhou, 730030 China; 9 Department of Orthopaedic Surgery, Xiangya Second Hospital of Central South University, Changsha, 410011 China; 10 Chongqing Key Laboratory for Oral Diseases and Biomedical Sciences, and the Affiliated Hospital of Stomatology of Chongqing Medical University, Chongqing, China; 11 Department of General Surgery, the Second Affiliated Hospital of Soochow University, Suzhou, 215004 China; 12 Department of Clinical Laboratory Medicine, the Affiliated Hospital of Qingdao University, Qingdao, 266061 China

## Abstract

Pancreatic ductal adenocarcinoma (PDAC) is one of the most deadly malignancies with <5% five-year survival rate due to late diagnosis, limited treatment options and chemoresistance. There is thus an urgent unmet clinical need to develop effective anticancer drugs to treat pancreatic cancer. Here, we study the potential of repurposing monensin as an anticancer drug for chemo-resistant pancreatic cancer. Using the two commonly-used chemo-resistant pancreatic cancer cell lines PANC-1 and MiaPaCa-2, we show that monensin suppresses cell proliferation and migration, and cell cycle progression, while solicits apoptosis in pancreatic cancer lines at a low micromole range. Moreover, monensin functions synergistically with gemcitabine or EGFR inhibitor erlotinib in suppressing cell growth and inducing cell death of pancreatic cancer cells. Mechanistically, monensin suppresses numerous cancer-associated pathways, such as E2F/DP1, STAT1/2, NFkB, AP-1, Elk-1/SRF, and represses EGFR expression in pancreatic cancer lines. Furthermore, the *in vivo* study shows that monensin blunts PDAC xenograft tumor growth by suppressing cell proliferation via targeting EGFR pathway. Therefore, our findings demonstrate that monensin can be repurposed as an effective anti-pancreatic cancer drug even though more investigations are needed to validate its safety and anticancer efficacy in pre-clinical and clinical models.

## Introduction

Pancreatic ductal adenocarcinoma (PDAC) is one of the most deadly diseases and one of the leading causes of cancer-related deaths in United States^1–4^. Most PDAC patients remain asymptomatic until the disease reaches an advanced stage^4^. In fact, only less than 20% of patients are present with localized, potentially resectable tumors^5^. As lifespan is being improved in general population, it is conceivable that the absolute case numbers of pancreatic cancer are likely to rise, especially in China, India and other Asian regions with large populations^6^. For example, in 2015 there were about 90,000 new cases diagnosed with PDAC and nearly 80,000 deaths due to this disease in China^7^. While multiple factors may contribute to the dismal prognosis for patients with pancreatic cancer, two notable clinical features of this disease may share the blame, late diagnosis and resistance to the already limited treatment options^2,8^. Despite decades of efforts, the overall five-year survival rate for pancreatic cancer remains at only ~5%^3,6,9^.

Even though the detailed tumorigenic mechanism behind PDAC remains to be fully elucidated, most pancreatic cancers arise from microscopic non-invasive epithelial proliferations within the pancreatic ducts^4^. Alterations of the four driver genes KRAS, CDKN2A, TP53, and SMAD4 are thought critical to the development of pancreatic cancer, in which KRAS mutation and alterations in CDKN2A are considered early events in pancreatic tumorigenesis^4^. A recent integrated genomic analysis of 456 PDAC samples has identified 32 recurrently mutated genes that aggregate into 10 pathways, including KRAS, TGF-β, WNT, NOTCH, ROBO/SLIT signaling, G1/S transition, SWI-SNF, chromatin modification, DNA repair and RNA processing^10^. Furthermore, transcriptomic analysis classified PDAC into 4 subtypes: squamous tumors, pancreatic progenitor tumors, immunogenic tumors, and aberrantly differentiated endocrine exocrine (ADEX) tumors, which correlate well with PDAC histopathological characteristics^10^. It is conceivable that such integrative genomic analysis of the molecular evolution of pancreatic cancer subtypes should identify potential targets for therapeutic development in the near future.

The past two decades have witnessed numerous progresses in the development of new and effective targeted cancer therapeutics. However, limited progress has been made in the drug development for pancreatic cancer due to its heterogeneity and drug resistance^9–11^. In most cases, surgical resection remains as the only potentially curative treatment, followed by post-operative adjuvant chemotherapy with gemcitabine or S-1, an oral fluoropyrimidine derivative^4^. FOLFIRINOX (fluorouracil, folinic acid, irinotecan, and oxaliplatin) and gemcitabine plus nanoparticle albumin-bound paclitaxel (nab-paclitaxel) are the treatments of choice for those who do not have surgery indications^4^. The use of gemcitabine in patients with advanced pancreatic cancer is associated with a significant, though marginal, survival extension of approximately one month^12^. Gemcitabine has been the cornerstone of PDAC treatment in all stages of the disease for the last two decades, but gemcitabine resistance develops within weeks of chemotherapy initiation^9^. The epithelial growth factor receptor (EGFR) inhibitor erlotinib is one of a few targeted agents that show promise in combination with gemcitabine although only achieving a marginal survival benefit in unselected patients^13^. Thus, it is urgent to develop effective anticancer drugs to treat pancreatic cancer.

The unmet need for more effective anticancer drugs has sparked a growing interest for drug repurposing, which involves in using drugs already approved for other indications to treat cancer^14^. Drug repurposing can also be a cost-effective alternative strategy to identify new small molecule-based therapies and may significantly influences the discovery of therapeutics although successful drug repurposing is challenging and subject to particular limitations^14,15^. Nonetheless, several of such repurposed anticancer drugs are currently in clinical trials^14–17^.

Here, we study the anticancer activity of an antibiotic, monensin, in human pancreatic cancer. As a polyether innophore antibiotic secreted by the bacteria *Streptomyce cinnamonensis*^18,19^, monensin has recently been shown to exhibit anti-proliferative effect on several cancer types. However, it is not clear whether monensin has similar anticancer effect on pancreatic cancer, particularly on gemcitabine-resistant pancreatic cancer. Using two human pancreatic cancer cell lines Panc-1 and MiaPaCa-2, we show that monensin suppresses cell proliferation and migration, and cell cycle progression, and induces cell death of gemcitabine-resistant human pancreatic cancer cells. Furthermore, monensin functions synergistically with gemcitabine or erlotinib to suppress cell growth and induce cell death of gemcitabine-resistant human pancreatic cancer cells. Mechanistic studies indicate that monensin targets several cancer-related signaling pathways and effectively inhibits EGFR expression in pancreatic cancer cell, which is elevated in PDAC samples. Lastly, monensin effectively blunts tumor growth *in vivo* and inhibits cell proliferation and EGFR expression in the xenograft tumors of gemcitabine-resistant PDAC cells. Collectively, these results demonstrate that monensin can be repurposed to treat pancreatic cancer, in particular for chemo-resistant PDAC, although further studies are required to validate its safety and anticancer efficacy in various pre-clinical and clinical models.

## Materials and Methods

### Cell culture and chemicals

Human pancreatic cancer cell line Panc-1 and MiaPaCa-2 were kindly provided by Dr. Keping Xie of the University of Texas MD Anderson Cancer Center. All cells were cultured in complete DMEM containing 10% fetal bovine serum (FBS, ThermoFisher, Waltham, MA) with 100 units/mL penicillin and 100 µg/mL streptomycin at 37 °C in 5% CO_2_ as previously described^20–23^. Monensin, gemcitabine and erlotinib were obtained from Cayman Chemical (Ann Arbor, MI) and LC Laboratories (Woburn, MA), respectively. Unless indicated, all other reagents were purchased from Sigma-Aldrich (St. Louis, MO) or Thermos Fisher Scientific (Waltham, MA).

### Crystal violet staining

Crystal violet staining was performed as previously described^24–27^. Briefly, subconfluent Panc-1 and MiaPaCa-2 were treated with the indicated concentrations of drugs (monensin, gemcitabine, erlotinib or drugs combinations). At 72 h post treatment, cells were washed, fixed and stained with 0.5% crystal violet/formalin for 30 min, followed by tape water rinse and air dry before taking macrographic images.

### WST-1 assay

Cell proliferation was measured by using Premixed WST-1 Reagent (Takara Bio USA, Mountain View, CA) as previously described^28–31^. Briefly, subconfluent Panc-1 and MiaPaCa-2 cells were plated in 96-well plates and treated with different concentrations of drugs (gemcitabine, monensin, erlotinib or drugs combinations) for 48 h. The Premixed WST-1 substrate was added to the wells, and incubated at 37 °C for 30 min, followed byreading at 440 nm using a microplate reader. Each assay condition was performed in triplicate.

### Cell wounding/migration assay

Cell wounding/migration experiments were carried out as previously described^32–36^. Specifically, cells were plated in 6-well culture plates to reach ~90% confluence. The monolayer cells were then scratched with pipette tips. At the indicated time points, wound healing status at the same locations was recorded. Each assay was set up in triplicate.

### Transwell cell migration analysis

Transwell assay was carried out as previously described^37–39^. Briefly, resuspended Panc-1 or MiaPaCa-2 cells were place in the upper chamber containing a layer of the 8 µm pore Corning transwell membrane (Millipore-Sigma) and treated with 4 µM monensin or DMSO control, while the lower chamber was filled with culture medium. At 12 h post treatment, the cells that migrated through the membrane were fixed, stained, and counted (e.g., 10 high-power fields were counted to determine the average migrated cells).

### Cell cycle analysis

Cell cycle analysis was carried out as previously described^29,40–43^. Specifically, Panc-1 and MiaPaCa-2 were plated in 6-well culture plates and treated with 2 µM monensin or solvent control. At 48 h, cells were harvested, fixed and stained with Magic Solution (10x stock: 0.5% NP-40, 3.4% formaldehyde, 10 ug/ml Hoechst 33258, in PBS) for 30 min, followed by flow cytometry using BD FACS Calibur-HTS. Data analysis was done with FlowJo v10.0 software. Each assay was performed in triplicate.

### Apoptosis analysis

The apoptosis analysis was determined by using the Annexin V staining assay as described^29,44–47^. Briefly, Panc-1 and MiaPaCa-2 were plated in 6-well cell culture plates and treated with different concentrations of monensin or vehicle control. At 48 h, cells were collected, resuspended in Annexin V Binding Buffer at 10^6^ cells/ml, and stained with Annexin V-FITC (BD Pharmingen, San Jose, CA) and propidium iodide (PI) for 15 min under a light-proof condition. The stained cells were subjected to flow cytometry using BD FACS Calibur-HTS. Data analysis was done with the FlowJo v10.0 Each assay was performed in triplicate.

### Chou-Talalay drug combination index determination

The drug combination effects between gemcitabine or EGFR inhibitor (erlotinib) and monensin was analyzed by using Chou-Talalay method^28,29,48,49^. Dose-dependent effects of each drug alone and in combinations on cell proliferation were first determined by WST-1 assay. The acquired data were calculated by using CompuSyn software (ComboSyn, Inc.). The obtained combination index (CI) from Chou-Talalay method provides a quantitative definition for additive effect (CI = 1), synergism (CI < 1), and antagonism (CI > 1) in different drug combinations as previously described^48,49^.

### Transfection and Gaussia luciferase assay

Gaussia luciferase (GLuc) reporter analysis was conducted as described^50–54^. A penal of cancer-associated signaling pathway reporters, such as E2F/DP1, Elk1/SRF, AP-1, NFκB, and STAT1/2 reporters, were homemade as previously described^29,33,52^. A constitutively active reporter pBG2Luc served as a control^29,33^. Briefly, Panc-1 cells were plated in 25 cm^2^ flasks at subconfluence and transfected with 3 µg/flask of different reporter plasmids using Lipofectamine according to the manufacturer’s instructions (Invitrogen). At 16 h post transfection, the transfected cells were reseeded into 24-well culture plates and treated with indicated concentrations of drug or vehicle control. At 24 h and 48 h post treatment, 50 µl of culture medium were taken for Gaussia luciferase assay using BioLux Gaussia Luciferase Assay Kit (New England Biolabs, NEB, Ipswich, MA). Each assay was carried out in triplicate.

### Total RNA purification and Touchdown-quantitative real-time PCR (TqPCR)

Subconfluent pancreatic cancer cells were treated with different concentrations of monensin for 48 h. RNA was isolated by using TRIZOL Reagents (Invitrogen) for reverse transcription with hexamer and M-MuLV reverse transcriptase (NEB). The cDNA products were used qPCR with the primers of the genes of interest designed with Primer3 program (Supplementary Table S1)^55^. TqPCR was done by using SYBR Green-based qPCR on a CFX-Connect unit (Bio-Rad Laboratories, Hercules, CA) as previously described^50,56–58^. Each qPCR condition was done in triplicate. *GAPDH* was used to normalize gene expression levels.

### Immunofluorescence staining

The immunofluorescence staining was performed as previously reported^59–61^. Briefly, Panc-1 and MiaPaCa-2 were exposed to various concentrations of drug or vehicle control. At 36 h, the cells were fixed and immunofluorescence stained with an anti–EGFR antibody (Santa Cruz Biotechnology, Santa Cruz, CA). Negative control was set up by incubating the cells with control IgG.

### Xenograft tumors of human pancreatic cancer lines

Animal experiments were approved by the Institutional Animal Care and Use Committee at The University of Chicago. All experimental procedures were performed by following the approved protocol. Xenograft tumor model was established as previously described^28,29,39,43,62–65^. Briefly, Panc-1 cells were stably tagged with FLuc (firefly luciferase) (Panc-1/FLuc) using the piggyBac system^30,41,60^. Exponentially growing Panc-1/FLuc cells were harvested, prepared in sterile PBS at 10^7^cells/ml and subcutaneously injected into the flanks of nude mice (ENVIGO, 6–8 week-old, male, 3.0 × 10^6^ cells perinjection, 6 sites permouse, n = 5 per group). After 3 days, the mice were randomly divided into two groups: monensin (10 mg/kg, i.p., once every 36 h) or vehicle control (i.p., once every 36 h). Tumor masses were monitored by caliper measurement and whole body bioluminescence imaging with the Xenogen IVIS 200 Imaging System at 7, 11, 16, 26 days post treatment. All mice were euthanized after4 weeks. Tumor masses were harvested.

### H & E staining and immunohistochemical (IHC) staining

The use of clinical pancreatic cancer samples and normal pancreatic samples was approved by the Institutional Ethic Committee of West China Hospital, Sichuan University. The archived pancreatic samples were delinked without the patients’ identifiers and used for IHC with the waived informed consent according to the U. S. NIH’s guidelines involving human subjects. A total of 8 cases of pancreatic cancer samples and 6 cases of non-tumor pancreatic samples were obtained from the Department of Pancreatic Surgery of West China Hospital at Sichuan University. The tumor samples recovered from the xenograft model were also subjected to H & E staining as previously described^25,66,67^.

The IHC staining was conducted as previously reported^37,68–70^. Briefly, paraffin-embedded tissue sections were processed with deparaffinization, rehydration, and antigen retrieval, followed by IHC staining with an anti-EGFR or anti-PCNA antibody (Santa Cruz Biotechnology). Negative controls were set up by incubating the slides with control IgGs.

### Statistical analysis

All quantitative experiments were either repeated three times independently and/or performed in triplicate. The student’s *t* test and one-way analysis of variance were used to calculate the statistical significance, which was defined as *p* < 0.05.

## Results

### Monensin suppresses cell proliferation and migration of gemcitabine-resistant pancreatic cancer cells

**Figure 1:**
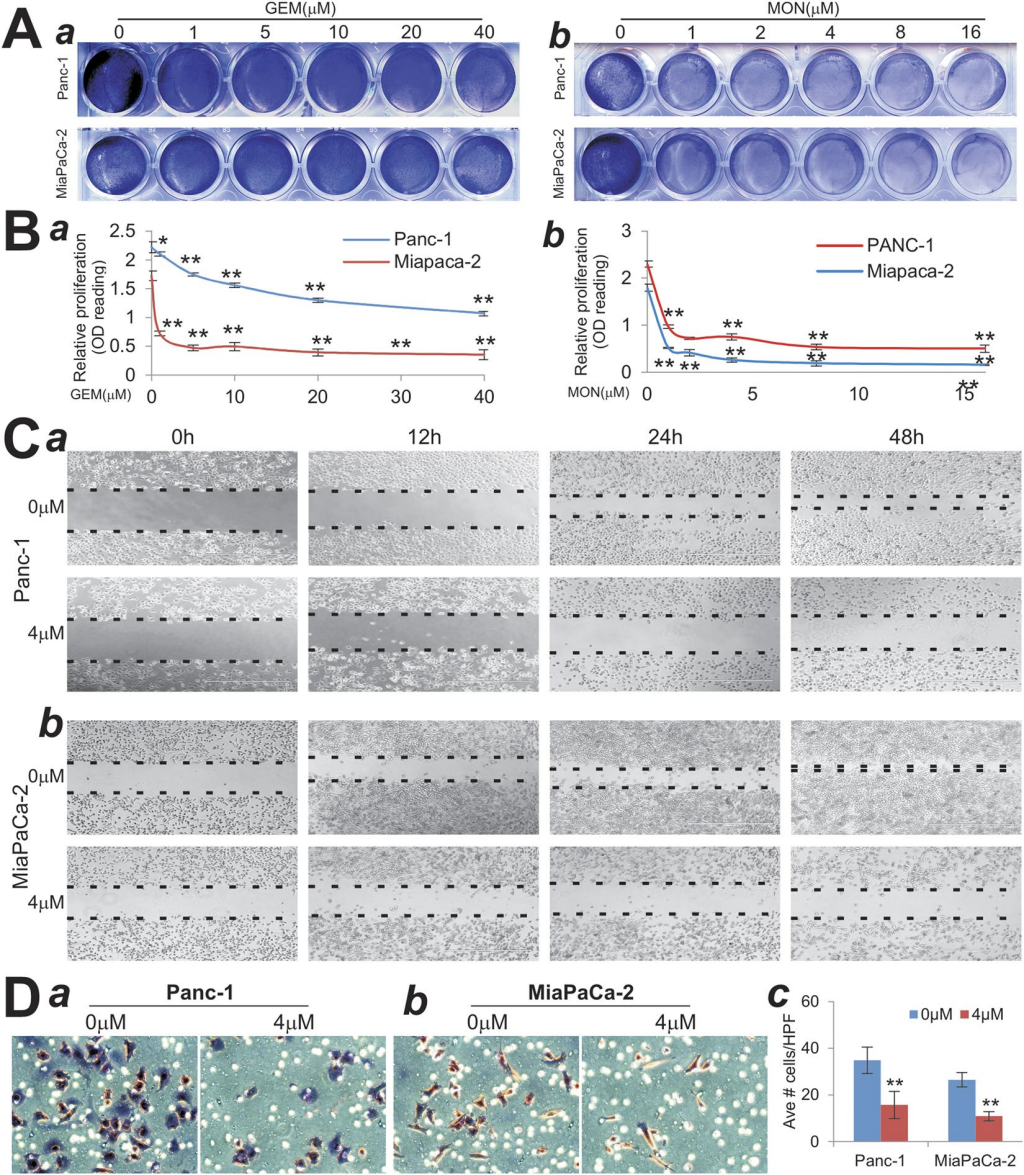
Monensin effectively inhibits cell proliferation and migration of gemcitabine-resistant pancreatic cancer cells. (**A**) Crystal violet staining assay. Subconfluent Panc-1 and MiaPaCa-2 cells were treated with gemcitabine (GEM) (*a*) or monensin (MON) (*b*) at the indicated concentrations. At 72 h post treatment, the cells were fixed and stained with crystal violet. Representative results are shown. (**B**) WST-1 assay. Panc-1 and MiaPaCa-2 cells were seeded in 96-well plates and treated with varied concentrations of gemcitabine (GEM) or monensin (MON). At 24 h (*a*) or 48 h (*b*) WST-1 reagent was added to plates and incubated for 30 min, and absorbance measurement was performed. All assay conditions were done in triplicate. “*”p < 0.05 and “**”p < 0.01, compared with that of the control groups. (**C**) Cell wound healing assay. Exponentially growing Panc-1 (*a*) and MiaPaCa-2 (*b*) cells were wounded with micro-pipette tips and treated with monensin at indicated concentrations. The gaps were recorded at 0 h, 12 h, 24 h, 36 h and 48 h after treatment. The dotted lines indicate the edge of the wound. Each assay condition was done in triplicate. Representative results are shown. (**D**) Transwell cell assay. Resuspended Panc-1 (*a*) and MiaPaCa-2 (*b*) cells were treated with 0 or 4 µM monensin, the cells migrated through the membrane were fixed, stained and quantitatively determined (*c*). Representative images are shown. “**”p < 0.01, compared with that of the control groups.

We first tested the effect of gemcitabine on two commonly-used human pancreatic cancer cell lines Panc-1 and MiaPaCa-2. Gemcitabine is one of the first-line chemotherapy agents for pancreatic cancer. When subconfluent Panc-1 and MiaPaCa-2 cells were exposed to escalating concentrations of gemcitabine, crystal violet staining demonstrated that Panc-1 cells were resistant to gemcitabine and survived well at an even high concentration of 40 µM, although MiaPaCa-2 cells were slightly more sensitive to gemcitabine (Fig. 1A, panel a). However, under the same growth condition monensin was shown to effectively suppress cell proliferation of both lines at a concentration as low as 1 µM (Fig. 1A, panel b), suggesting that monensin may act as a potent anticancer agent for pancreatic cancer cells.

We further carried out the WST-1 cell proliferation assays and confirmed the above findings. Specifically, Panc-1 cells were inhibited ineffectively by gemcitabine and showed about only 20% inhibition even at 80 µM of gemcitabine, while MiaPaCa-2 cells were inhibited by 80% at 20 µM of gemcitabine (Fig. 1B, panel a). On the other hand, the WST-1 assay results showed that the cell proliferation was drastically suppressed by monensin at a concentration of as low as 0.5 µM monensin for both Panc-1 (p < 0.01) and MiaPaCa-2 (p < 0.01) (Fig. 1B, panel b). Taken together, the above results indicate that monensin can effectively suppress pancreatic cancer cell proliferation and overcome gemcitabine resistance in pancreatic cancer cells, particularly in Panc-1 cells.

### Monensin suppresses cell wound healing and migration of pancreatic cancer cells

We next tested whether monensin treatment impacts wound healing and cell migration of pancreatic cancer cells. When confluent Panc-1 and MiaPaCa-2 monolayer cells were scratched and added with 0 or 4 uM monensin, we found that the rate of wound closure was significantly lower in monensin-treated Panc-1 (Fig. 1C, panel a) and MiaPaCa-2 (Fig. 1C, panel b) than that of the control group (or 0 µM monensin) at all examined the time points. For example, at 48 h, the wound gap was only ~20% and ~5% in Panc-1 and MiaPaCa-2 cells, respectively, compare to the starting time point (or 0 h) in the control groups. However, in the presence of 4 µM monensin the rate of wound closure in Panc-1 and MiaPaCa-2 was significantly reduced, and approximately 90% of the wound remained open in both cell lines (Fig. 1C, panels a vs. b).

We conducted the transwell cell migration assay to assess the effect of monensin on cell migration capability of pancreatic cells. While both Panc-1 and MiaPaCa-2 cells were shown to migrate through the transwell membrane rather effectively (Fig. 1D, panels a,b), the presence of monensin (at 4 µM) significantly reduced the average cell numbers migrated through the transwell membrane in both cell lines (p < 0.01) (Fig. 1D, panel c). Collectively, the above findings strongly suggest that, in addition to its ability to inhibit cell proliferation, monensin may significantly diminish the cell wound healing and migration capabilities of pancreatic cancer cells.

### Monensin suppresses cell cycle progression and induces apoptosis in human pancreatic cancer cells

**Figure 2:**
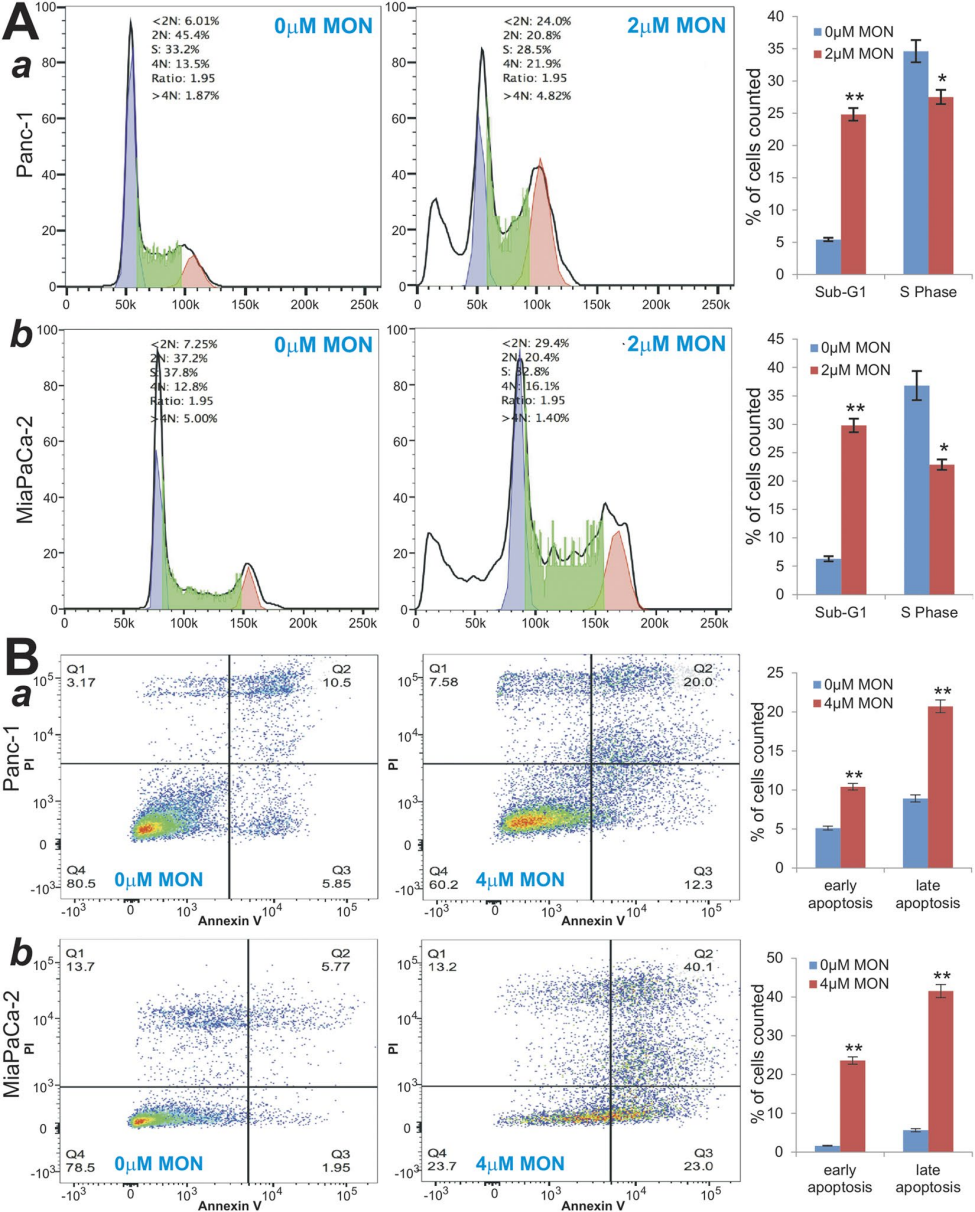
Monensin inhibits cell cycle progression and induces apoptosis in human pancreatic cancer cells. (**A**) Cell cycle analysis. Exponentially growing Panc-1 (*a*) and MiaPaCa-2 (*b*) cells were treated with monensin (2 µM) or vehicle control for 48 h. Cells were collected and stained, then subjected to FACS analysis. Percentages of cells in sub-G1 phase and S phase were graphed. “*” p < 0.05 and “**” p < 0.01, compared with that of the control groups. (**B**) Annexin-V apoptosis assay. Panc-1 (*a*) and MiaPaCa-2 (*b*) cells were treated with monensin (4 µM) or vehicle control. At 48 h post treatment, cells were collected and stained with Annexin V-FITC and propodium iodide, and subjected to flow cytometry. Average percentages of apoptotic cells (including early apoptosis and late apoptosis) were calculated and graphed. “**” p < 0.01, compared with that of the control groups.

To uncover possible mechanism through which monensin inhibits pancreatic cancer cell proliferation, we analyzed the cell cycle profile in monensin-treated pancreatic cancer cells and found a drastic increase in the sub-G1 phase and a remarkable decrease in S phase of Panc-1 and MiaPaCa-2 cells, compared with that of the control groups (Fig. 2A, panels a,b), consistent with monensin’s ability to inhibit cell proliferation of pancreatic cancer cells. Notably, the G1 phase in both lines also significantly decreased, which may be caused by the marked increase in sub-G1 populations.

Furthermore, Annexin-V based apoptosis analysis demonstrated that monensin was able to induce both early apoptosis and late apoptosis, compared with that of the control groups (Fig. 2B, panels a,b). For example, in Panc-1 cells, the percentage of early apoptotic cells and late apoptotic cells in the 4 µM monensin group were 12% and 20%, respectively, compared with 5.85% and 10.5% in the control group (p < 0.01) (Fig. 2B, panel a). Similarly, in MiaPaCa-2 cells the percentage of early apoptotic cells and late apoptotic cells in the 4 µM monensin group were 23% and 40.1%, respectively, compared with 1.95% and 5.77% in control group (p < 0.01) (Fig. 2B, panel b). We also assessed the nucleus morphologic evidence of monensin-induced apoptosis in pancreatic cancer cells through Hoechst 33258 staining and found that monensin (at 4 µM for 48 h) induced significantly higher % of nuclear condensation and DNA fragmentation in Panc-1 and MiaPaCa-2 cells, compared with that of the control groups (data not shown). Therefore, the above findings suggest monensin may suppress pancreatic cancer cell proliferation at least in part by inhibiting cell cycle progression and inducing apoptosis.

### Monensin acts synergistically with gemcitabine or EGFR inhibitor erlotinib on suppressing cell growth and inducing cell death of human pancreatic cancer lines

**Figure 3:**
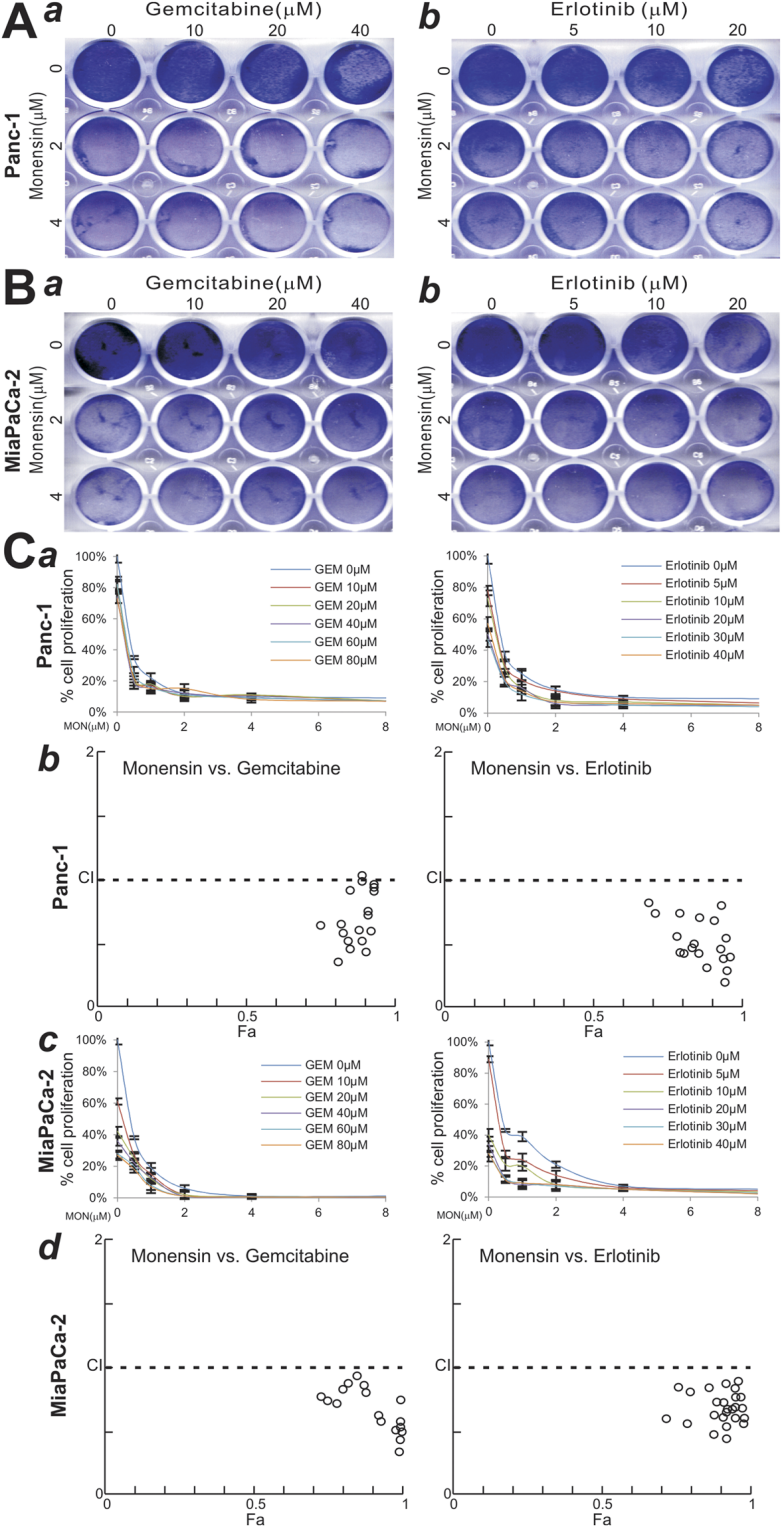
Monensin acts synergistically with gemcitabine or erlotinib on inhibiting cell proliferation of human pancreatic cancer cells. (**A,B**) Crystal violet assay for drug combinations. Panc-1 (**A**) and MiaPaCa-2 (**B**) cells were treated with monensin/gemcitabine combination (*a*) or monensin/erlotinib combination (*b*) at the indicated concentrations. At 72 h post treatment, cells were fixed and stained with crystal violet. Representative results are shown. (**C**) WST-1 assay for drug combinations and Chou-Talalay drug combination index analysis. Panc-1 (*a*,*b*) and MiaPaCa-2 (*c*,*d*) cells were treated with monensin/gemcitabine combination or monensin/erlotinib combination at indicated concentrations (*a*,*c*). At 48 h post treatment, WST-1 reagent was added to each well and incubated for 30 min. WST-1 activities were measured and graphed. The WST-1 assay data were further calculated for the combination index (CI) using the Chou-Talalay method (*b*,*d*). CI < 1, synergistic effect; CI = 1, additive effect; and CI > 1, antagonistic effect.

We tested if monensin would synergize with and/or sensitize pancreatic cancer cells to the currently used first-line chemotherapeutic drugs, such as gemcitabine and EGFR targeted inhibitor erlotinib, to inhibit pancreatic cancer cell proliferation. Qualitative crystal violet staining assay showed that, when subconfluent Panc-1 cells were co-treated with gemcitabine or erlotinib and different concentrations of monensin, menonsin was shown to enhance the inhibitory effects of gemcitabine (Fig. 3A, panel a) or erlotinib (Fig. 3A, panel b) in a dose-dependent fashion. Similar results were obtained in MiaPaCa-2 cells (Fig. 3B, panels a,b). The above findings indicate that monensin can potentiate the inhibitory effects that gemcitabine or erlotinib exerts on pancreatic cancer cells.

We also carried out the quantitative WST-1 assays on drug combinations (Fig. 3C, panels a,c). Using the data acquired from the WST-1 assays, we analyzed the combination index (or CI) with the well-established Chou-Talalay method^48,49^. We found the CI values for both monensin/gemcitabine and monensin/erlotinib combinations in Panc-1 cells were <1, indicating that these combinations may exhibit synergistic effects (Fig. 3C, panel b). Similarly, in MiaPaCa-2 cells the CI values for both monensin/gemcitabine and monensin/erlotinib combinations were also <1, indicating that these combinations may exhibit synergistic effects (Fig. 3C, panel d).

**Figure 4:**
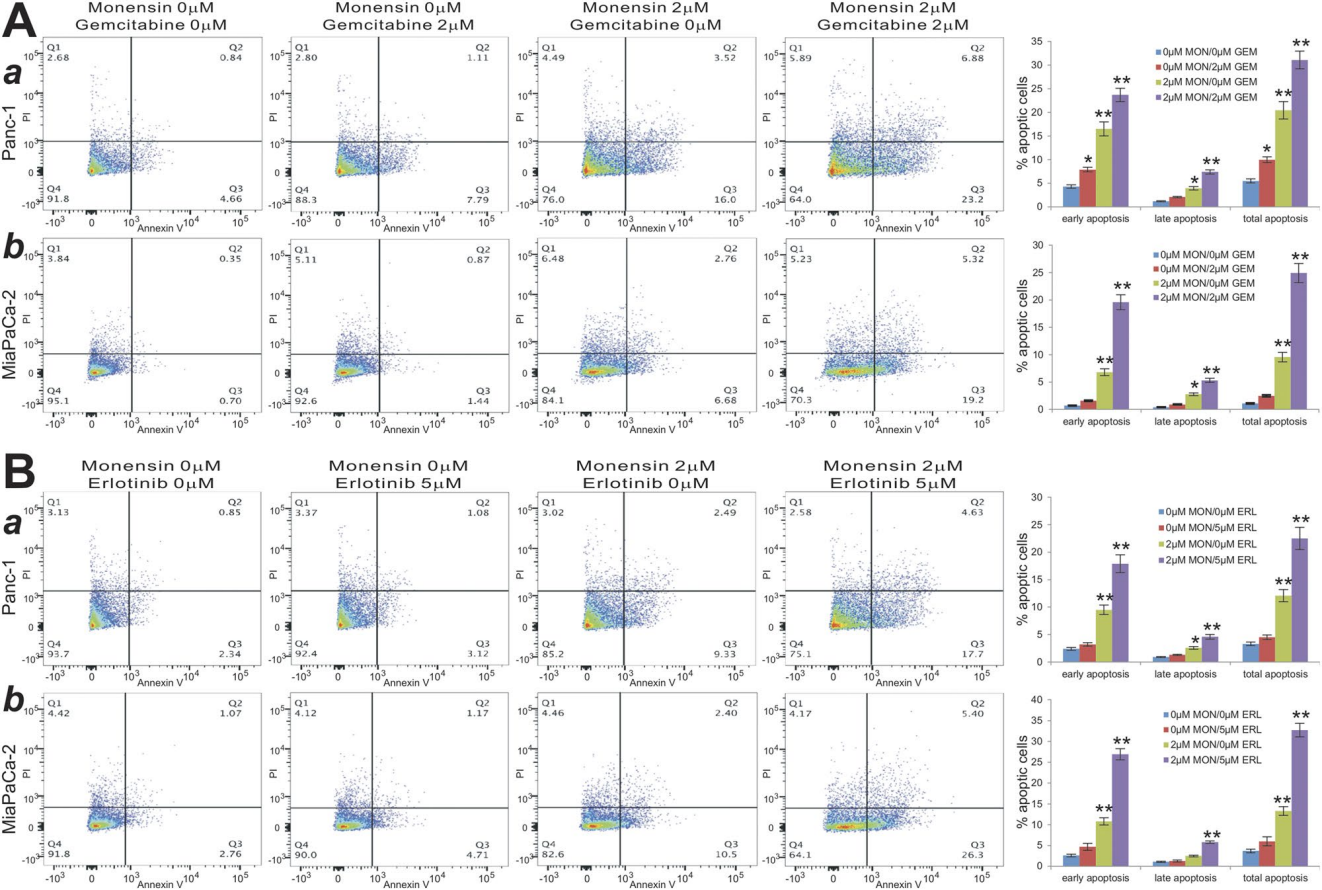
Monensin potentiates gemcitabine or erlotinib-induced apoptosis of human pancreatic cancer cells. Monensin/gemcitabine combinations (**A**) or monensin/erlotinib combinations (**B**) at the indicated concentrations were carried out in Panc-1 (*a*) and MiaPaCa-2 (*b*). At 48 h post treatment, cells were collected, stained with Annexin V-FITC and propodium iodide, and subjected to flow cytometry. Average percentages of apoptotic cells were calculated and graphed. “*” p < 0.05 and “**” p < 0.01, compared with that of the control groups.

Furthermore, we analyzed the effect of combinations of monensin and gemcitabine on inducing apoptosis in both Panc-1 and MiaPaCa-2 cells, and found that monensin significantly enhanced gemcitabine-induced early and late phases of apoptosis (Fig. 4A, panels a,b). Similarly, monensin significantly augmented erlotinib-induced early and late phases of apoptosis (Fig. 4B, panels a,b). Collectively, these findings strongly demonstrate that monensin can synergize with gemcitabine or erlotinib on inhibiting the cell growth and inducing cell death of pancreatic cancer cells.

### Monensin suppressed several cancer-associated pathways and effectively inhibits the expression of EGFR in pancreatic cancer cells

**Figure 5:**
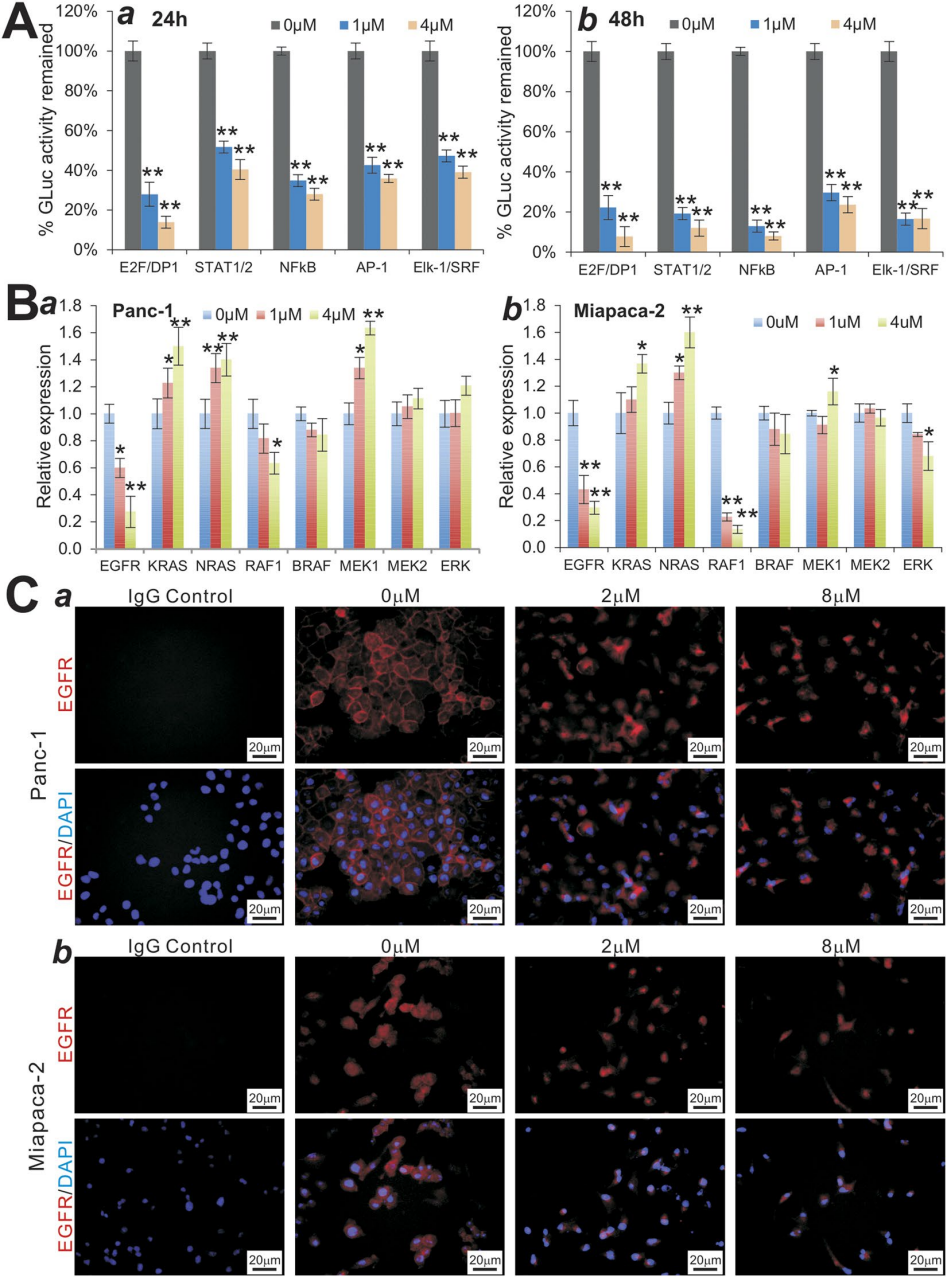
Monensin inhibits multiple cancer-associated pathways and targets EGFR signaling in human pancreatic cancer cells. (**A**) Effect of monensin on several important cancer-associated pathways. Subconfluent Panc-1 cells were transfected with the indicated GLuc reporter plasmids and treated with monensin at the indicated concentrations. At 24 h (*a*) and 48 h (*b*) post treatment, culture media were collected for Gaussia luciferase activity assay. Each assay condition was done in triplicate. “**” p < 0.01, compared with that of the control groups. (**B**) Effect of monensin on EGFR signaling pathway. Subconfluent Panc-1 (*a*) and MiaPaCa-2 (*b*) cells were treated with the indicated concentrations of monensin for 48 h. Total RNA was isolated and subjected to TqPCR analysis of the expression of EGFR and related genes. Human GAPDH was used as the reference gene. “*” p < 0.05 and “**” p < 0.01, compared with that of the control groups. (**C**) Effect of monensin on EGFR protein level. Subconfluent Panc-1 (*a*) and MiaPaCa-2 (*b*) cells were treated with monensin at the indicated concentrations or vehicle control. At 36 h, cells were fixed and subjected to immunofluorescence staining with an EGFR antibody. The cell nuclei were counter-stained with DAPI. Control IgG was used as a negative control. Representative results are shown.

To explore the potential mechanisms through which monensin exerts anticancer effects on pancreatic cancer cells, we surveyed the effects of monensin on a panel of five well-characterized cancer-associated signal pathway reporters as we previously described^28,29,33,45,52,71,72^. When the Gaussia luciferase reporters were introduced into Panc-1 cells and treated with 0, 1 µM or 4 µM monensin for 24 h and 48 h, the Gaussia luciferase activities for the E2F/DP1, STAT1/2, NFκB, AP-1 and Elk-1/SRF reporters were significantly inhibited (p < 0.01) (Fig. 5A, panels a,b). It is noteworthy that we also analyzed the reporter activities for NFAT, HIF1A, RBP-JK, MYC/MAX, TCF/LEF, CREB, and TGFB/SMAD pathways and found their activities were not significantly affected by monensin.

Based on the results from the reporter assays and the previous reports from ours and other labs^19,29^, we speculated that monensin might target EGFR pathway. Thus, we analyzed the expression levels of the EGFR and EGFR-regulated downstream genes following monensin treatment in Panc-1 and MiaPaCa-2 cells. Using TqPCR^56^, we found that the expression of EGFR and RAF1 was significantly repressed in both cell lines (Fig. 5B, panels a,b). Interestingly, KRAS and NRAS, and to a lesser extent MEK1, were shown to be significantly up-regulated by monensin, especially at 4 µM level (Fig. 5B, panels a,b). While we do not have any satisfactory explanations about such up-regulations, it is conceivable the up-regulation may be caused by negative feedback inhibitions upon monensin treatment.

We also examined the effect of monensin on EGFR expression at protein level. Subconfluent Panc-1 and MiaPaCa-2 were treated with varied concentrations of monensin. EGFR expression was examined by immunofluorescence analysis. We found that while the membrane-bound and/or whole cell expression of EGFR was readily detected in the control or vehicle treated cells, EGFR expression, especially at cell membrane, was significantly suppressed by monensin, although there was significant nuclear stainging upon monensin treatment (Fig. 5C, panel a,b). Nonetheless, the above results further indicate monensin can target EGFR signaling in pancreatic cancer.

**Figure 6:**
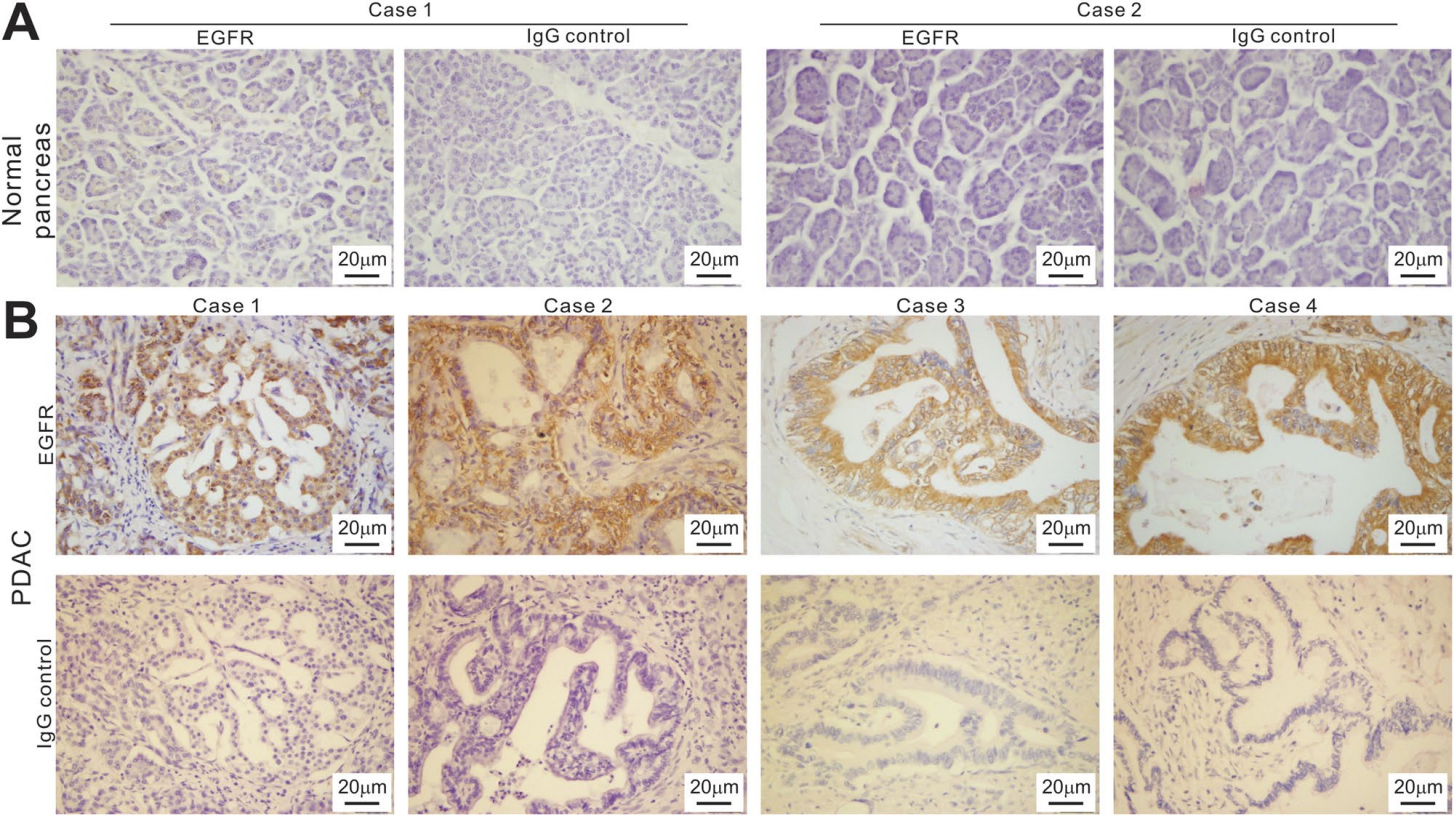
EGFR is highly expressed in human pancreatic cancer tissues. Two cases of normal pancreatic tissues (**A**) and four cases of representative pancreatic cancer samples (**B**) tissues were sectioned and subjected to immunohistochemical staining with an anti-EGFR antibody. Control IgG was used as a negative control. Representative images are shown.

We further analyzed EGFR expression in the clinical samples of pancreatic cancer. While EGFR expression in normal pancreatic samples was not apparently detectable, seven of the examined eight cases of pancreatic cancer samples exhibited strong EGFR staining in the cancerous ductal cells (Fig. 6A vs. B). The remaining samples had weaker but detectable EGFR expression in cancerous regions (data not shown). Taken together, these results demonstrate that EGFR may be targeted by monensin, which may at least in part explain how monensin exerts its effective anticancer activity against pancreatic cancer cells.

### Monensin effectively blunts the tumor growth and inhibits cell proliferation and EGFR expression in the xenograft model of human pancreatic cancer *in vivo*

**Figure 7:**
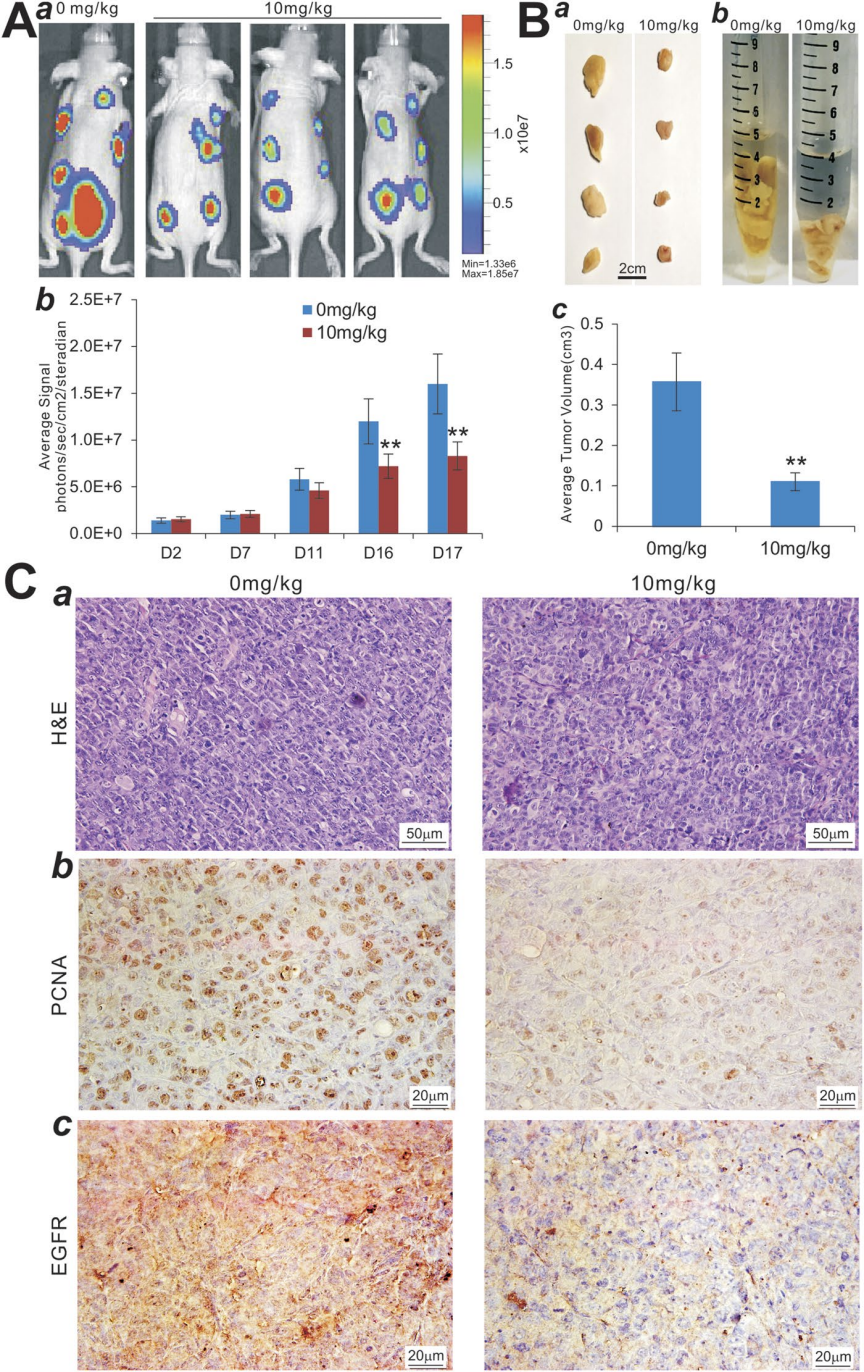
Monensin blunts the tumor growth and inhibits cell proliferation and EGFR expression in the xenograft model of human pancreatic cancer cells *in vivo*. (**A**) Xenogen bioluminescence imaging of xenograft tumor growth. Firefly luciferase-labeled Panc-1 cells were subcutaneously injected into athymic nude mice and randomly divided into two groups. At 3 days post injection, the animals were treated with monensin (10 mg/kg) or vehicle control. The mice were imaged at 7, 11, 16, 26 days after treatment, and sacrificed at 4 weeks of injection. Representative images at day 26 are shown (*a*). The average signal for each group at different time points was calculated and graphed (*b*). (**B**) Retrieved tumor samples and average tumor volume. Representative gross image of the retrieved tumors (*a*) and accumulative tumor masses from each group (*b*) are shown. The average tumor volume for each group at the endpoint was also calculated (*c*). “**” p < 0.01, compared with that of the control group. (**C**) Histologic evaluation and immunohistochemical staining. The retrieved tumor samples from each group were fixed, paraffin-embedded and subjected to H & E staining (*a*) and immunohistochemical staining using anti-PCNA (*b*) or anti-EGFR antibody (*c*). Control IgGs were used as negative controls. Representative images are shown.

Lastly, we examined the *in vivo* anticancer activity of monensin in the xenograft tumor model of human pancreatic cancer. The firefly luciferase-tagged Panc-1 cells were first injected into the flanks of athymic mice. After 3 days, the mice were randomly divided into two groups and treated with monensin (10 mg/kg body weight) or vehicle control. Tumor progression was monitored through whole body Xenogen bioluminescence imaging (Fig. 7A panel a). Xenogen imaging data analysis indicates that monensin effectively suppressed tumor growth at as early as 11 days after treatment, compared with the control group (Fig. 7A, panel b). At 4 weeks after treatment, the tumor masses recovered from the control group are significantly larger, either in individual tumors or bulk tumor volumes, than that of the monensin treatment group (Fig. 7B, panels a–c). These findings further confirm that monensin can effectively suppress the tumor growth of gemcitabine-resistant pancreatic cancer cells *in vivo*.

Nonetheless, histologic evaluation of the tumor masses retrieved from both groups did not show significant differences (Fig. 7C, panel a). However, IHC analysis indicated that the expression of the cell proliferation marker PCNA dramatically decreased in the tumor samples retrieved from the monensin treatment group, compared with that of the control group (Fig. 7C, panel b). Similarly, immunohistochemical staining indicated that the EGFR expression was significantly diminished in the tumor samples retrieved from the monensin treatment group, compared with that of the control group (Fig. 7C, panel c), consistent with the possibility that EGFR may be targeted by monensin in pancreatic cancer cells. Collectively, the *in vitro* and *in vivo* findings strongly demonstrate that monensin exerts a potent inhibitory effect on cell proliferation and tumor growth in drug-resistant pancreatic cancer cells, possibly through targeting the EGFR signaling pathway.

## Discussion

Pancreatic cancer ranks fourth among cancer related deaths, and the disappointing five-year survival rate of below 5% results from drug resistance to all known therapies, as well as from disease presentation at a late stage when PDAC is already metastatic^5,9^. Most PDAC patients suffer from recurrence within 24 months and die of the progressively worsening treatment-resistant cancer. Recently, major treatment breakthroughs in many difficult-to-treat cancers, such as melanoma, have been facilitated by the identification of actionable mutant oncogenic driver genes^73^. While there has been an increasing understanding of the underlying biology of pancreatic cancer, unfortunately, until now, there are no actionable therapeutic targets for PDAC. Meanwhile, only modest improvement in effective systemic chemotherapy has been attained in pancreatic cancer. Therefore, pancreatic cancer is still one of the most lethal cancers with a dismal 5-year survival less than 5%^5^. Thus, there is an unmet clinical need to develop more effective and safe treatment for the clinical management of PDAC patients. The repurposing of existing non-cancer drugs represents a cost-effective alternative to develop new treatment options for cancer patients with high unmet medical needs. In this study, we demonstrate that monensin may be repurposed to treat chemo-resistant pancreatic cancer. Our results suggest the monensin may act synergistically with gemcitabine or erlotinib in combination chemotherapy for the treatment of drug-resistant pancreatic cancer.

Monensin was discovered as a polyether innophore antibiotic over half century ago^18,19^. It has a rather favorable biosafety profile as monensin is widely used in cattle and poultry feed^18,19,29^. It was reported that malignant cells were approximately 20 times more sensitive to monensin than normal cells^74^. We and others showed that monensin exhibits anti-proliferative effects on several other types of cancer, including renal cancer, lung cancer, colon cancer, myeloma, prostate cancer, and ovarian cancer cells^19,29,74–80^. In this study, we further demonstrate that monensin exerts potent anticancer activity in chemo-resistant pancreatic cancer cells by inhibiting its proliferation, cell cycle progression, and cell migration and by inducing apoptosis. Moreover, monensin acts synergistically with gemcitabine or erlotinib to inhibit cell proliferation and induce apoptosis in chemo-resistant pancreatic cancer cells. Our *in vivo* study in xenograft tumor model of PDAC cells further validates the biosafety and anticancer efficacy of monensin as a repurposed anti-PDAC agent.

Mechanistically, monensin may accomplish its anticancer effect by targeting multiple signaling pathways, particularly the EGFR signaling pathway. Given the fact that monensin exerts its anti-proliferation effect in chemo-resistant pancreatic cancer cells at very low micromole concentrations when compared with gemcitabine or erlotinib. While more mechanism-based studies are needed, it was reported that monensin can reduce the expression of cyclin A, CDK6, and cyclin D1while inducing programmed cell death-related genes, such as caspase-3, caspase-8, Bax, and mitochondria transmembrane potential in some types of human cancer lines^76–80^. It has been recently shown that monensin can suppress Wnt signaling in colorectal cancer cells^81^, and EGFR signaling in ovarian cancer cells^29^.

In this study, we also investigated the effect of monensin on multiple cancer-related pathways and found that monensin can inhibit E2F/DP1, STAT1/2, NFκB, AP-1 and Elk-1/SRF pathways. Furthermore, the expression of EGFR and its downstream genes, such as RAF1 and BRAF, is effectively suppressed by monensin. The above findings suggest that monensin may exert its potent proliferation suppression effect through the inhibition of multiple growth factor-induced signal pathways, especially EGFR, which is found overexpressed in the clinical samples of pancreatic cancer. Interestingly, it was reported that monensin may impact the endocytic recycling pathway of EGFR^19,82^. We further demonstrate that the EGFR expression level in the PDAC xenograft tumors was significantly inhibited by monensin. Therefore, our findings strongly demonstrate that monensin can exert its potent anticancer activity in chemo-resistant PDAC cells at least in part by targeting the EGFR signaling pathway. It is conceivable that, even though both target EGFR, the mode of action for monensin should be distinct from that of erlotinib’s since monensin can synergize with erlotinib in PDAC cells. Collectively, our findings suggest monensin can be repurposed to treat pancreatic cancer, although its safety and anticancer efficacy need to be further validated in preclinical and clinical studies.

## Electronic supplementary material

Suppl Table 1S
