# Supplementary material for: Monensin inhibits cell proliferation and tumor growth of chemo-resistant pancreatic cancer cells by targeting the EGFR signaling pathway
Source: Sci Rep. 2018 Dec 17;8:17914. doi: 10.1038/s41598-018-36214-5 (PMC6297164; doi:10.1038/s41598-018-36214-5)
Supplement: Supplementary file 1 — Suppl Table 1S [file 41598_2018_36214_MOESM1_ESM.docx]

**Monensin inhibits cell proliferation and tumor growth of chemo-resistant pancreatic cancer cells by targeting the EGFR signaling pathway**

**Running Title**: **Repurposing monensin as an anti-pancreatic cancer agent**

Xin Wang^1,2^, Xingye Wu^2,3^, Zhonglin Zhang^2,4^, Chao Ma^2,4^, Tingting Wu^2,4^, Shengli Tang^2,4^, Zongyue Zeng^2,5^, Shifeng Huang^2,3^, Cheng Gong^2,4^, Chengfu Yuan^2,6^, Linghuan Zhang^2,5^, Yixiao Feng^2,3^, Bo Huang^2,5,7^, Wei Liu^2,3^, Bo Zhang^2,8^, Yi Shen^2,9^, Wenping Luo^2,10^, Xi Wang^2,5^, Bo Liu^2,3^, Yan Lei^2,3^, Zhenyu Ye^2,11^, Ling Zhao^2,3^, Daigui Cao^2,5^, Lijuan Yang^2,8^, Xian Chen^2,12^, Rex C. Haydon^2^, Hue H. Luu^2^, Bing Peng^1^, Xubao Liu^1,^* and Tong-Chuan He^2^*

^Materials and Methods:
email:
, West China Hospital, Sichuan University. 610041, Chengdu, China.1^ Materials and Methods:
email:
, West China Hospital, Sichuan University. 610041, Chengdu, China.DMaterials and Methods:
email:
, West China Hospital, Sichuan University. 610041, Chengdu, China.epartment of Pancreatic Surgery, West China Hospital of Sichuan University, Chengdu 610041, China;

^2^ Molecular Oncology Laboratory, Department of Orthopaedic Surgery and Rehabilitation Medicine, The University of Chicago Medical Center, Chicago, IL 60637, USA

^3^ Departments of Surgery, Clinical Laboratory Medicine, Orthopaedic Surgery, Plastic Surgery and Burn, Otolaryngology, Head and Neck Surgery, and Obstetrics and Gynecology, the First Affiliated Hospital of Chongqing Medical University, Chongqing 400016, China

^4^ Departments of Hepatobiliary & Pancreatic Surgery, Neurosurgery, and Otolaryngology, Head and Neck Surgery, the Affiliated Zhongnan Hospital of Wuhan University, Wuhan 430071, China

^5^ Ministry of Education Key Laboratory of Diagnostic Medicine and School of Laboratory Medicine, and the Affiliated Hospitals of Chongqing Medical University, Chongqing 400016, China

^6^ Department of Biochemistry and Molecular Biology, China Three Gorges University School of Medicine, Yichang 443002, China

^7^ Department of Clinical Laboratory Medicine, the Second Affiliated Hospital of Nanchang University, Nanchang 330031, China

^8^ Key Laboratory of Orthopaedic Surgery of Gansu Province, and the Departments of Orthopaedic Surgery and Obstetrics and Gynecology, the First and Second Hospitals of Lanzhou University, Lanzhou, 730030, China

^9^ Department of Orthopaedic Surgery, Xiangya Second Hospital of Central South University, Changsha 410011, China

^10^ Chongqing Key Laboratory for Oral Diseases and Biomedical Sciences, and the Affiliated Hospital of Stomatology of Chongqing Medical University, Chongqing, China

^11^ Department of General Surgery, the Second Affiliated Hospital of Soochow University, Suzhou 215004, China

^12^ Department of Clinical Laboratory Medicine, the Affiliated Hospital of Qingdao University, Qingdao 266061, China

* Corresponding authors

**CORRESPONDENCES**

Xubao Liu, MD, PhD

DMaterials and Methods:
email:
, West China Hospital, Sichuan University. 610041, Chengdu, China.epartment of Pancreatic Surgery

West China Hospital

Sichuan University

Chengdu, China.

E-mail: [xbliu@medmail.com.cn](mailto:xbliu@medmail.com.cn)

T.-C. He, MD, PhD

Molecular Oncology Laboratory

The University of Chicago Medical Center

5841 South Maryland Avenue, MC 3079

Chicago, IL 60637, USA

Tel. (773) 702-7169

Fax (773) 834-4598

E-mail: [tche@uchicago.edu](mailto:tche@uchicago.edu)

**Supplemental Table S1. List of qPCR Primers**

| **Supplemental Table 1. List of qPCR Primers** | | |
| --- | --- | --- |
| Gene | Forward | Reverse |
| EGFR | CCAAGGGAGTTTGTGGAGAA | CTTCCAGACCAGGGTGTTGT |
| KRAS | TGTGGTAGTTGGAGCTGGTG | TGACCTGCTGTGTCGAGAAT |
| NRAS | GACTCGTGGTTCGGAGGC | ACCAAGGAGCGGCACTTC |
| RAF1 | CACCCTGCCTGTGGACAG | TGGTGCCCGCTCTCTTTG |
| BRAF | TAAGATGGCGGCGCTGAG | CTCCGGAATGGCAGGGTC |
| MEK1 | TGCAGGTTGGCTCTGCTC | AGGAGGCCCAAAAGCGAC |
| MEK2 | CGCTCCTACATGGCTCCG | TCCAGCTCTTTGGCGTCG |
| ERK1 | CCAGACCATGATCACACAGG | CTGGAAAGATGGGCCTGTTA |
| GAPDH | CAGCGACACCCACTCCTC | TGAGGTCCACCACCCTGT |
